# 2-[(4-Chloro­phen­yl)selan­yl]-3,4-di­hydro-2*H*-benzo[*h*]chromene-5,6-dione: crystal structure and Hirshfeld analysis

**DOI:** 10.1107/S2056989017007605

**Published:** 2017-05-31

**Authors:** Julio Zukerman-Schpector, Karinne E. Prado, Luccas L. Name, Rodrigo Cella, Mukesh M. Jotani, Edward R. T. Tiekink

**Affiliations:** a Departmento de Química, Universidade Federal de São Carlos, 13565-905 São Carlos, SP, Brazil; b Departamento de Engenharia Química, Centro Universitário da FEI, 09850-901, São Bernardo do Campo, São Paulo, Brazil; c Department of Physics, Bhavan’s Sheth R. A. College of Science, Ahmedabad, Gujarat 380 001, India; d Centre for Crystalline Materials, School of Science and Technology, Sunway University, 47500 Bandar Sunway, Selangor Darul Ehsan, Malaysia

**Keywords:** crystal structure, selenium, pyran derivative, C—Cl⋯π inter­actions, Hirshfeld surface analysis

## Abstract

The title organoselenium compound, C_19_H_13_ClO_3_Se {systematic name: 2-[(4-chloro­phen­yl)selan­yl]-2*H*,3*H*,4*H*,5*H*,6*H*-naphtho­[1,2-*b*]pyran-5,6-dione}, has the substituted 2-pyranyl ring in a half-chair conformation with the methyl­ene-C atom bound to the methine-C atom being the flap atom. The dihedral angle between the two aromatic regions of the mol­ecule is 9.96 (9)° and indicates a step-like conformation. An intra­molecular Se⋯O inter­action of 2.8122 (13) Å is noted. In the crystal, π–π contacts between naphthyl rings [inter-centroid distance = 3.7213 (12) Å] and between naphthyl and chloro­benzene rings [inter-centroid distance = 3.7715 (13) Å], along with C—Cl⋯π(chloro­benzene) contacts, lead to supra­molecular layers parallel to the *ab* plane, which are connected into a three-dimensional architecture *via* methyl­ene-C—H⋯O(carbon­yl) inter­actions. The contributions of these and other weak contacts to the Hirshfeld surface is described.

## Abstract 2

A step-like conformation about the pyranyl ring is found for the mol­ecular structure of the title compound. The three-dimensional packing is sustained by π–π, C—Cl⋯π and C—H⋯O inter­actions.

## Chemical context   

The natural product, β-lapachone (see Scheme) can be isolated from the bark of the lapacho tree found in Central and South American countries (see: http://www.beta-lapachone.com/). It exhibits biological activities in the context of cancer (Park *et al.* 2014 ▸), being known to induce apoptotic cell-death pathways in a number of cancer cell lines, including breast cancer (Schaffner-Sabba *et al.*, 1984 ▸), leukaemia (Chau *et al.*, 1998 ▸) and prostate cancer (Li *et al.*, 1995 ▸). In an allied application, β-lapachone can be used as a sensitizer in radiotherapy on prostrate (Suzuki *et al.*, 2006 ▸) and colon (Kim *et al.*, 2005 ▸) cancer cells.

Compounds of the bio-essential element selenium, found in amino acids such as seleno­cysteine and seleno­methio­nine, are known to hold potential as pharmaceutical agents (Tiekink, 2012 ▸), including in the realm of anti-cancer drugs (Seng & Tiekink, 2012 ▸). A key aspect of developing metal-based drugs is to incorporate a heavy element into the structure of a biologically active organic mol­ecule and with this in mind, it was thought of inter­est to attempt to incorporate selenium into the structure of β-lapachone. This was attempted by reacting lawsone, paraformaldehyde and (4-chloro­phen­yl)(ethen­yl)selane, as detailed in *Synthesis and crystallization*. Two major products were isolated, *i.e.* derivatives of α-lapa­chone and β-lapachone. The latter, hereafter (I), could be crystallized and was subjected to an X-ray structure determ­ination along with an analysis of its Hirshfeld surface in order to obtain more information on the mol­ecular packing. The results of this study are reported herein.
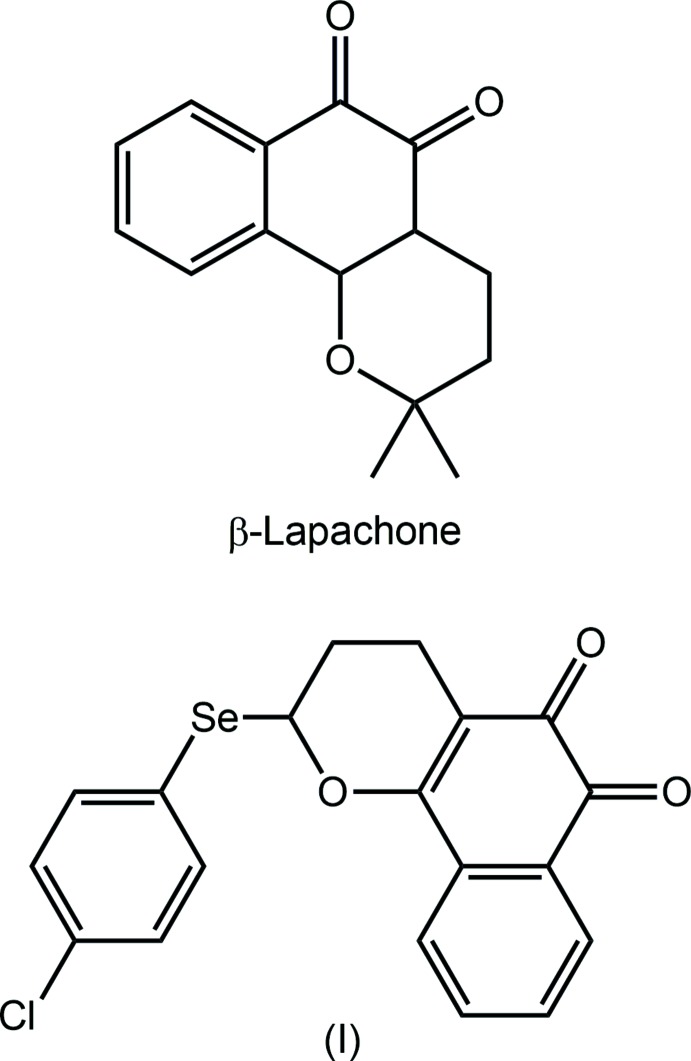


## Structural commentary   

**Figure 1:**
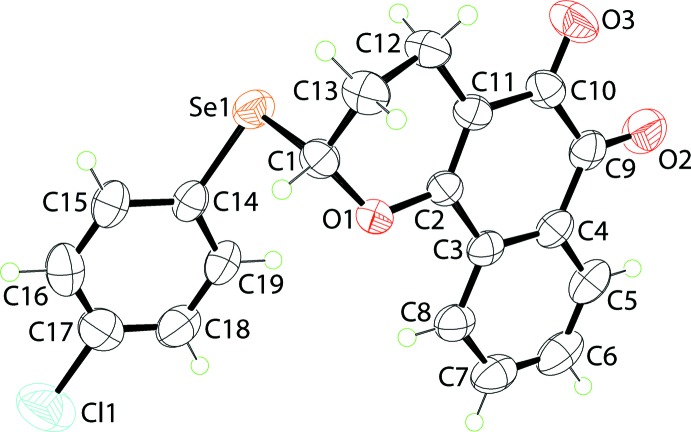
The mol­ecular structure of (I), showing the atom-labelling scheme and displacement ellipsoids at the 50% probability level.

The substituted 2-pyranyl ring in (I) (Fig. 1 ▸) adopts a half-chair conformation with the C13 atom lying 0.620 (3) Å above the plane through the remaining five atoms (r.m.s. deviation = 0.0510 Å). The 12 atoms comprising the naphthalene-1,2-dione ring system are almost coplanar, with an r.m.s. deviation of 0.0152 Å. This plane forms a dihedral angle of 9.96 (9)° with the chloro­benzene ring bound to the selanyl atom, indicating a near parallel disposition and a step-like arrangement between the aromatic substituents about the 2-pyranyl ring. An intra­molecular Se⋯O inter­action of 2.8122 (13) Å is noted; this observation is discussed further in the *Database survey*.

## Supra­molecular features   

**Figure 2:**
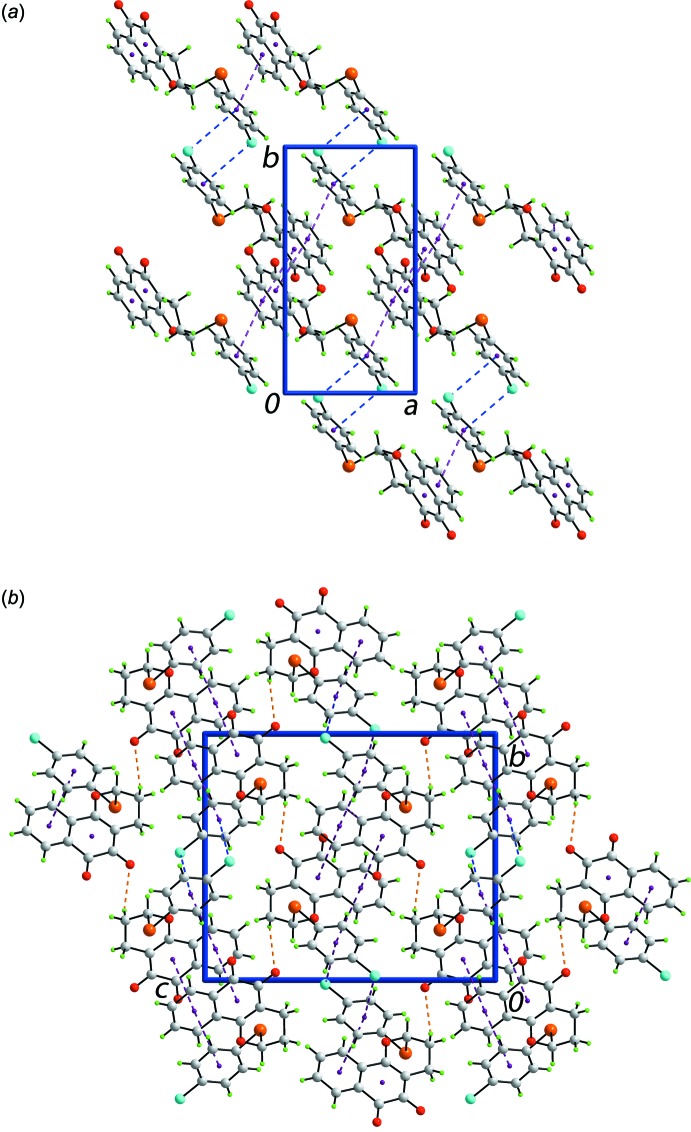
The mol­ecular packing in (I), showing (*a*) a view of the supra­molecular layer sustained by π–π and C—Cl⋯π inter­actions and (*b*) a view of the unit-cell contents in projection down the *a* axis. The π–π, C—Cl⋯π and C—H⋯O inter­actions are shown as purple, blue and orange dashed lines, respectively.

In the mol­ecular packing of (I), both rings of the naphthyl residues of centrosymmetrically related mol­ecules form close π–π contacts, *i.e. Cg*(C2–C4/C9–C11)⋯*Cg*(C3–C8)^i^ = 3.7213 (12) Å for an angle of inclination = 0.72 (9)° and symmetry operation (i) −*x*, −*y*, −*z*. Two types of inter­actions connect centrosymmetric aggregates into a supra­molecular layer parallel to the *ab* plane (Fig. 2 ▸ *a*). Thus, π–π inter­actions between naphthyl and chloro­benzene rings are formed, [*Cg*(C3–C8)⋯*Cg*(C14–C19)^ii^ = 3.7715 (13) Å with an angle of inclination = 9.95 (10)° and symmetry operation (ii) −1 + *x*, *y*, *z*] along with C—Cl⋯π(chloro­benzene) contacts between centrosymmetrically related rings (Table 1 ▸). Connections between layers are of the type methyl­ene-C—H⋯O(carbon­yl) (Table 1 ▸) to consolidate the three-dimensional packing (Fig. 2 ▸ *b*).

## Hirshfeld surface analysis   

**Figure 3:**
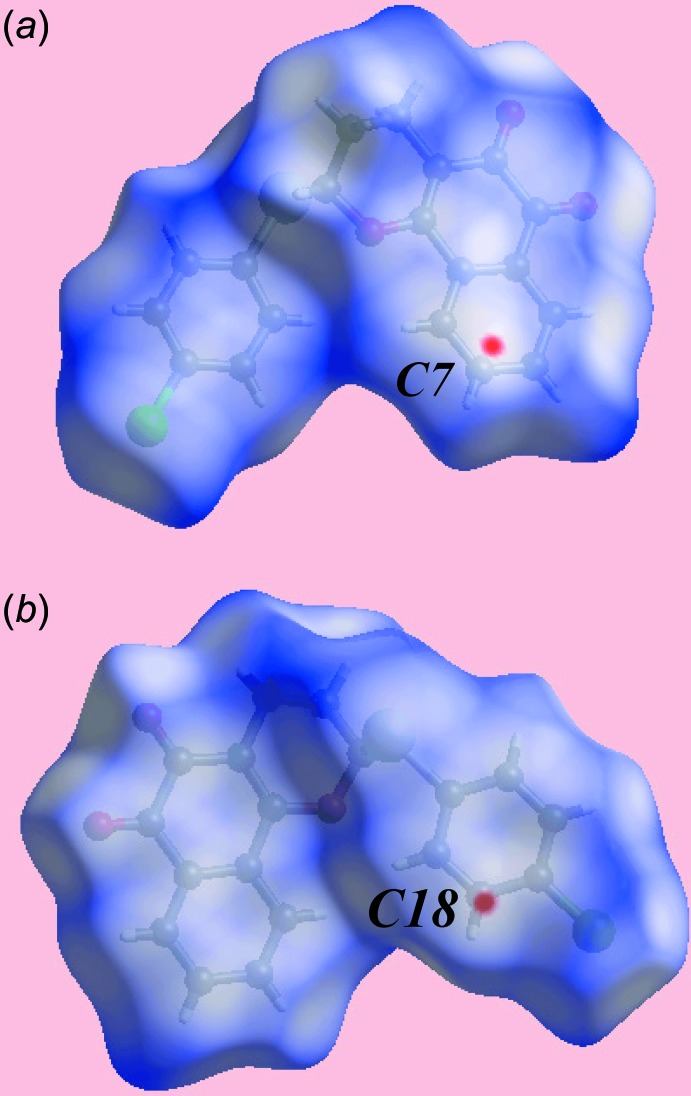
Two views of the Hirshfeld surface for (I) plotted over *d*_norm_ in the range −0.032 to 1.401 au.

**Figure 4:**
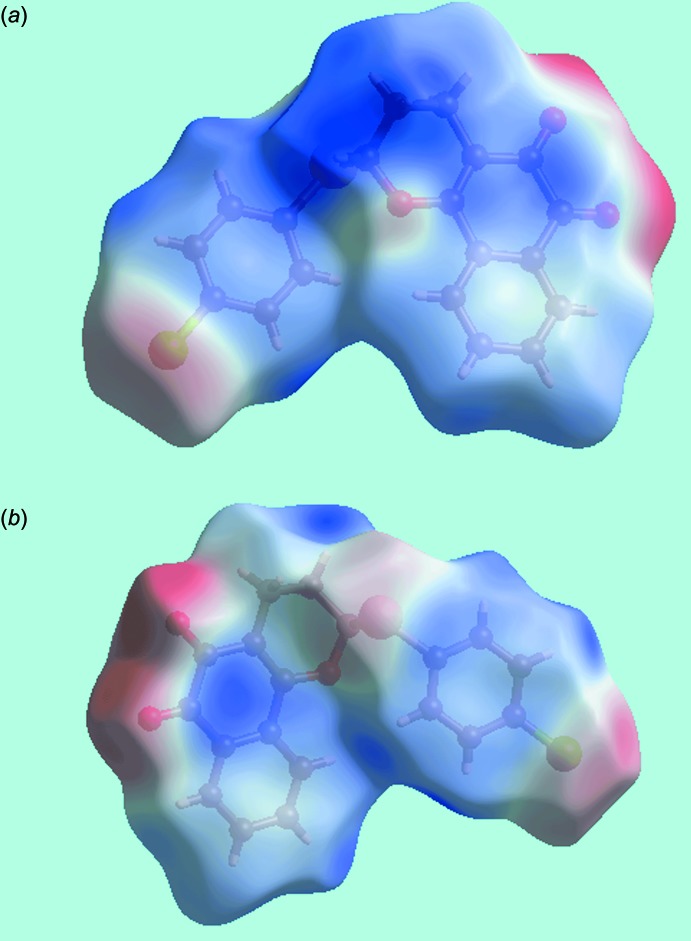
A view of Hirshfeld surface for (I) mapped over the calculated electrostatic potential in the range −0.067 to + 0.039 au. The red and blue regions represent negative and positive electrostatic potentials, respectively.

**Figure 5:**
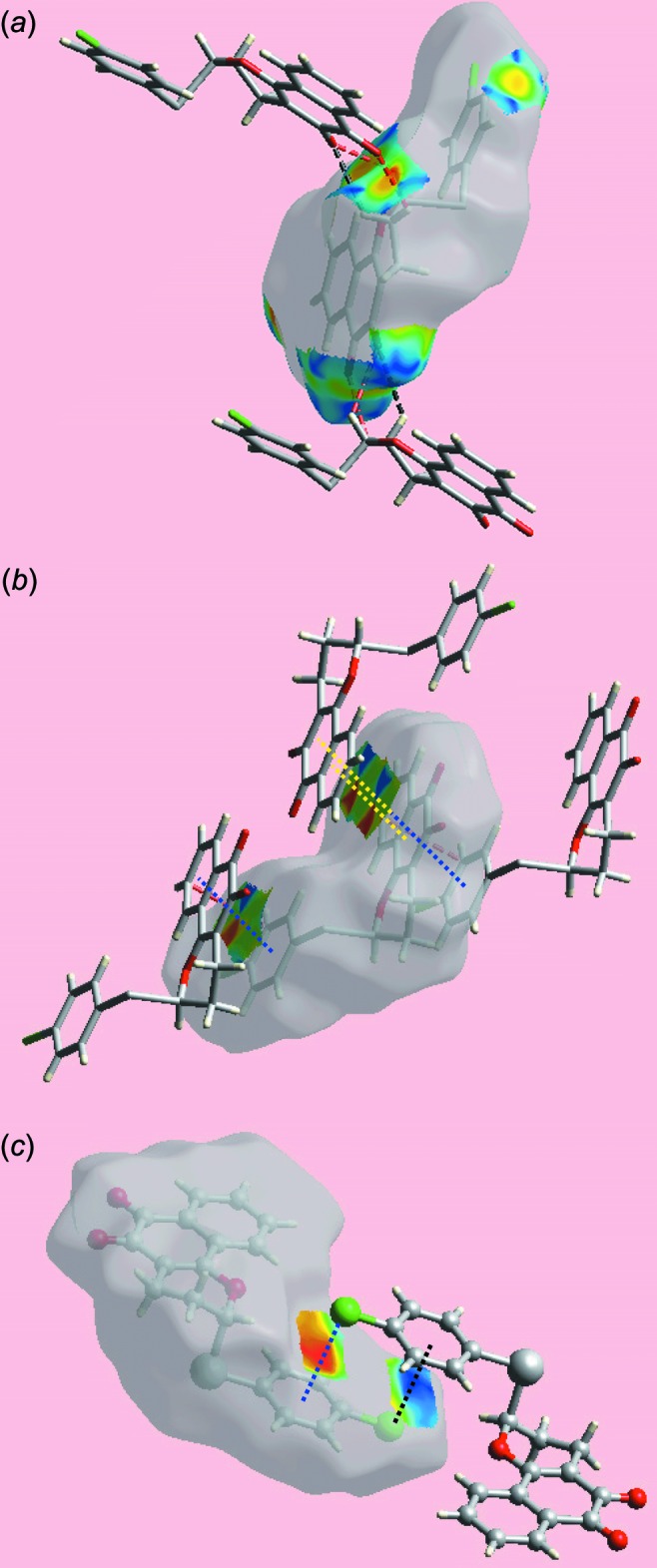
Views of Hirshfeld surfaces mapped over the shape-index about a reference mol­ecule, showing (*a*) C—H⋯O and short inter­atomic O⋯H/H⋯O contacts by black and red dashed lines, respectively, (*b*) π–π stacking inter­actions between naphthyl residues and between chloro­benzene and naphthyl rings by blue and yellow dotted lines, respectively and (*c*) C—Cl⋯π/π⋯Cl—C stacking contacts between chloro­benzene rings with black and blue dotted lines.

The Hirshfeld surfaces calculated on the structure of (I) also provide insight into the inter­molecular inter­actions; the calculation was performed as in a recent publication (Jotani *et al.*, 2016 ▸). The presence of bright-red spots appearing near the naphthyl-C7 and phenyl-C18 atoms on the Hirshfeld surface mapped over *d*_norm_ in Fig. 3 ▸ are due to a short inter­atomic C⋯C contact (see Table 2 ▸), significant in the crystal of (I). The absence of characteristic red spots near other atoms on the *d*_norm_-mapped Hirshfeld surface confirms the absence of conventional hydrogen bonds in the structure except for a weak C—H⋯O inter­action as given in Table 1 ▸. The blue and red regions corresponding to positive and negative electrostatic potentials on the Hirshfeld surface mapped over electrostatic potential, in Fig. 4 ▸ are the result of polarization of charges localized near the atoms. The immediate environments about a reference mol­ecule within shape-index-mapped Hirshfeld surfaces highlighting inter­molecular C—H⋯O inter­actions, short inter­atomic O⋯H/H⋯O contacts, π–π stacking inter­actions and C—Cl⋯π contacts are illustrated in Fig. 5 ▸.

**Figure 6:**
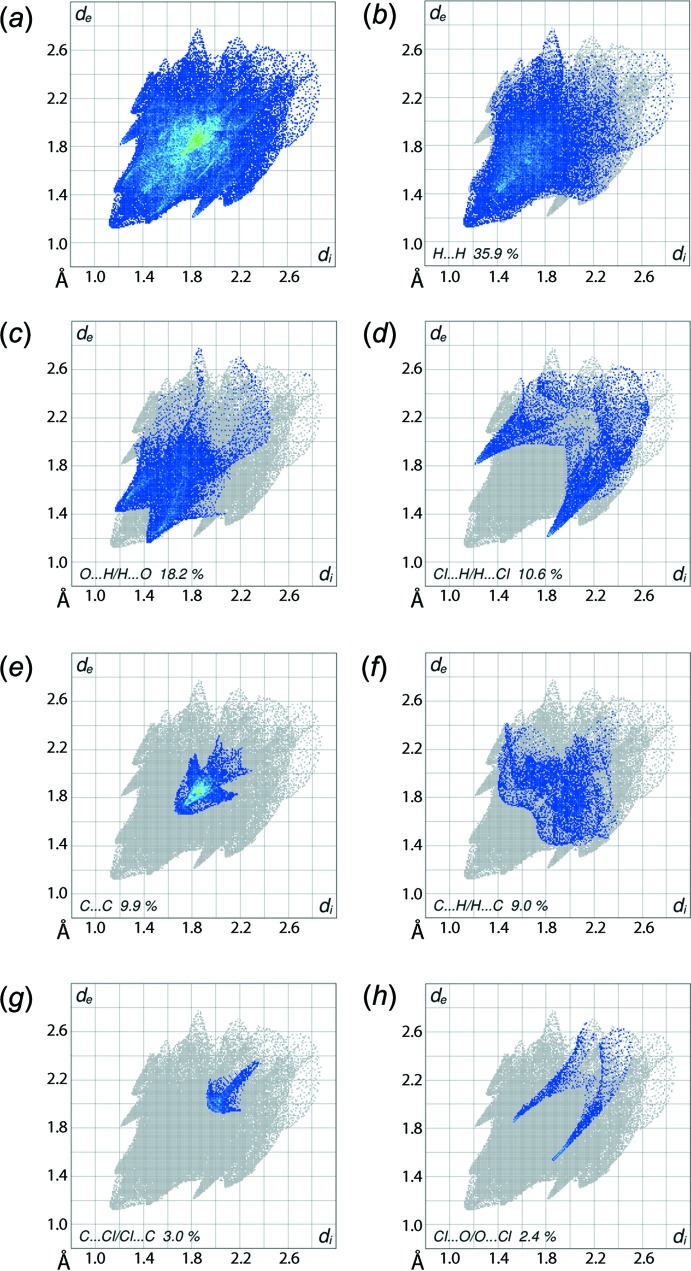
(*a*) The full two-dimensional fingerprint plot for (I) and fingerprint plots delineated into (*b*) H⋯H, (*c*) O⋯H/H⋯O, (*d*) Cl⋯H/H⋯Cl, (*e*) C⋯C, (*f*) C⋯H/H⋯C, (*g*) C⋯Cl/Cl⋯C and (*h*) Cl⋯O/O⋯Cl contacts.

The overall two-dimensional fingerprint plot (Fig. 6 ▸ *a*) and those delineated into H⋯H, O⋯H/H⋯O, Cl⋯H/H⋯Cl, C⋯C, C⋯H/H⋯C, C⋯Cl/Cl⋯C and Cl⋯O/O⋯Cl contacts (McKinnon *et al.*, 2007 ▸) are illustrated in Fig. 6 ▸ *b*–*h*, respectively; the relative contributions from the various contacts to the Hirshfeld surfaces are summarized in Table 3 ▸. The relatively low, *i.e*. 35.9%, contribution from H⋯H contacts to the Hirshfeld surface of (I) is due to the low content of hydrogen atoms in the mol­ecule and the involvement of some hydrogen atoms in short inter­atomic O⋯H/H⋯O contacts (Tables 1 ▸ and 2 ▸). The single peak at *d*_e_ + *d*_i_ ∼2.3 Å in Fig. 6 ▸ *b* is the result of a short inter­atomic H⋯H contact (Table 2 ▸). The inter­molecular C—H⋯O inter­action in the crystal is recognized as the pair of peaks at *d*_e_ + *d*_i_ ∼2.6 Å in the O⋯H/H⋯O delineated fingerprint plot (Fig. 6 ▸ *c*); the points arising from the short inter­atomic O⋯H contacts are merged in the plot.

**Figure 7:**
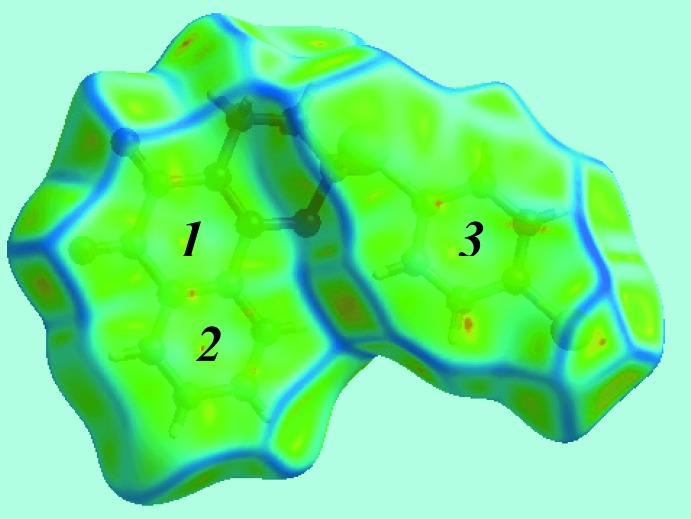
View of the Hirshfeld surface mapped over curvedness highlighting the flat regions corresponding to the C2–C4/C9–C11, C3–C8 and C14–C19 rings, labelled as 1, 2 and 3, respectively, involved in π–π stacking inter­actions.

The fingerprint plot delineated into C⋯C contacts, Fig. 6 ▸ *e*, characterizes the two π–π stacking inter­actions, one between inversion-related naphthyl rings, and the other between the chloro­benzene and (C2–C4/C9–C11) rings as the two overlapping triangular regions at around *d*_e_ = *d*_i_ ∼1.8 and 1.9 Å, respectively, having green points in the overlapping portion. The presence of these two π–π stacking inter­actions is also seen in the flat regions around the participating rings labelled with 1, 2 and 3 in the Hirshfeld surface mapped over curvedness in Fig. 7 ▸.

The chlorine atom on the benzene (C14–C19) ring makes a useful contribution to the mol­ecular packing. The small, *i.e*. 3.0%, contribution from C⋯Cl/Cl⋯C contacts (Fig. 6 ▸ *g*) to the Hirshfeld surface is the result of its involvement in a C—Cl⋯π contact formed between symmetry-related chloro­benzene atoms (Fig. 5 ▸ *c*). Its presence is also clear from the fingerprint plot delineated into Cl⋯H/H⋯Cl (Fig. 6 ▸ *d*), and Cl⋯O/O⋯Cl contacts (Fig. 6 ▸ *h*). The contribution from C⋯H/H⋯C contacts (Fig. 6 ▸ *f*) and other contacts (Table 3 ▸), including the selenium atom, have negligible influence on the packing as the inter­atomic separations are greater than sum of their respective van der Waals radii.
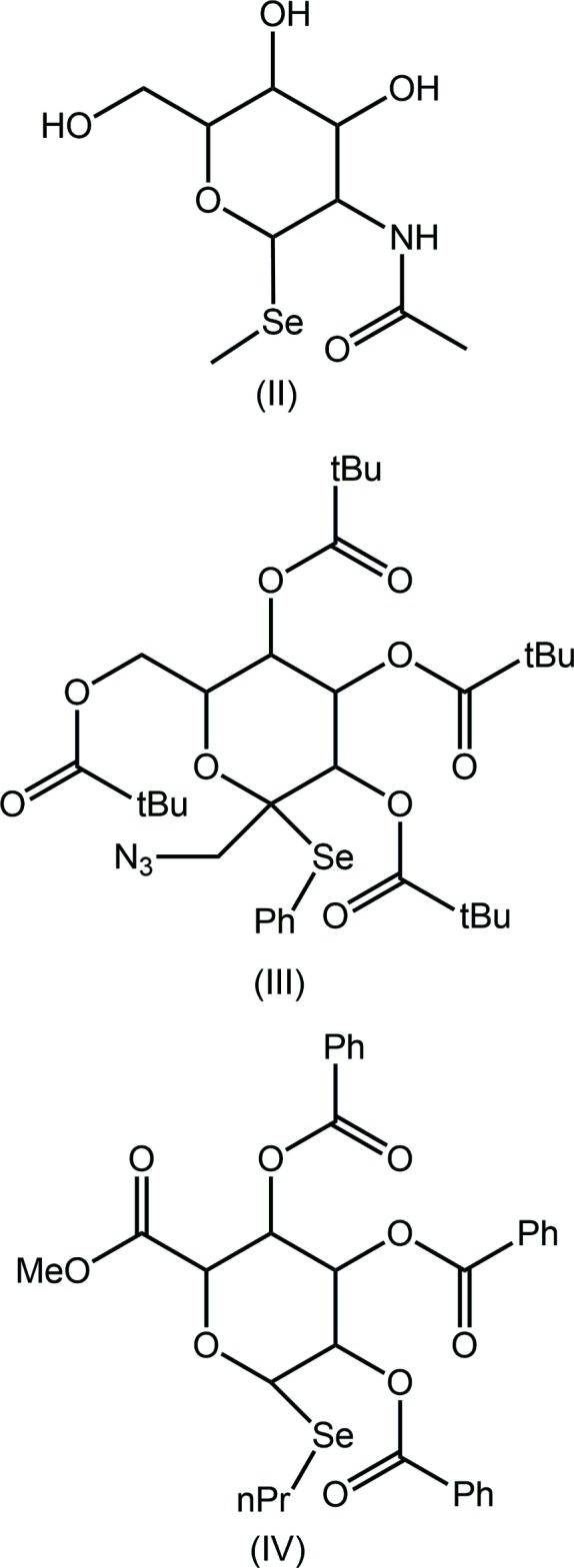


## Database survey   

There are three structures in the crystallographic literature (Groom *et al.*, 2016 ▸) having a similar 2-(organylselan­yl)oxane framework as in (I). The chemical diagrams for these, *i.e.* (II) (Traar *et al.*, 2004 ▸), (III) (Woodward *et al.*, 2010 ▸) and (IV) (McDonagh *et al.*, 2016 ▸) are shown in the Scheme above. Each of the structures features an intra­molecular Se⋯O inter­action as in (I). From the data collated in Table 4 ▸, there is no correlation between the Se⋯O distance and the C—Se—C angle, consistent with the weak nature of these inter­actions.

## Synthesis and crystallization   

Referring to the reaction scheme, in a double-necked flask equipped with a magnetic bar and reflux condenser, under a nitro­gen atmosphere, lawsone (1 mmol, 174 mg), paraformaldehyde (8 mmol, 240 mg), the vinyl selenide (1.5 mmol, 326 mg) and the ionic liquid 1-butyl-3-methyl­imidazolium chloride, [Bmim]Cl (1 mmol, 175 mg) were added over 1,4-dioxane (2 ml). The reaction mixture was heated at 383 K and stirred over 2 h. The reaction mixture was cooled and diluted with di­chloro­methane (100 ml) and then washed with water (3 × 50 ml). The organic phase was dried over Na_2_SO_4_, filtered and concentrated under vacuum. The crude product was purified in a silica gel-packed chromatography column, using ethyl acetate and hexane (2:8) as eluent to afford α-lapachone and β-lapachone (I) derivatives in 80% yield. Crystals of (I) were obtained by slow evaporation of a solvent mixture of hexane and ethyl acetate (8:2).
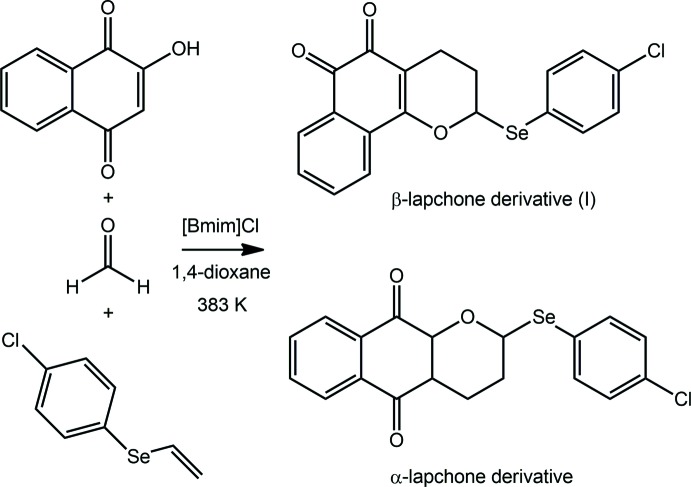


## Refinement details   

Crystal data, data collection and structure refinement details are summarized in Table 5 ▸. The carbon-bound H atoms were placed in calculated positions (C—H = 0.93–0.98 Å) and were included in the refinement in the riding-model approximation, with *U*_iso_(H) set to 1.2*U*_eq_(C).

## Supplementary Material

Crystal structure: contains datablock(s) I, global. DOI: 10.1107/S2056989017007605/wm5392sup1.cif

Structure factors: contains datablock(s) I. DOI: 10.1107/S2056989017007605/wm5392Isup2.hkl

Supporting information file. DOI: 10.1107/S2056989017007605/wm5392Isup3.cml

CCDC reference: 1551641

Additional supporting information:  crystallographic information; 3D view; checkCIF report

## Figures and Tables

**Table 1:** Hydrogen-bond geometry (Å, °) *Cg*1 is the centroid of the C14–C19 ring.

*D*—H⋯*A*	*D*—H	H⋯*A*	*D*⋯*A*	*D*—H⋯*A*
C13—H6⋯O3^i^	0.97	2.59	3.239 (2)	125
C17—Cl1⋯*Cg*1^ii^	1.74 (1)	3.72 (1)	4.000 (2)	86 (1)

Symmetry codes: (i) 
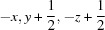
; (ii) 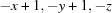.

**Table 2:** Summary of short inter­atomic contacts (Å) in (I)

Contact	distance	symmetry operation
H5⋯H11	2.27	*x*, 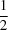 − *y*, 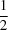 + *z*
O2⋯H5	2.70	-*x*, − 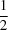 + *y*, 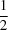 − *z*
O3⋯H9	2.70	-*x*, − 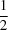 + *y*, 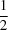 − *z*
C7⋯C18	3.346 (3)	−1 + *x*, *y*, *z*

**Table 3:** Percentage contributions of inter­atomic contacts to the Hirshfeld surfaces for (I)

Contact	percentage contribution
H⋯H	35.9
O⋯H/H⋯O	18.2
Cl⋯H/H⋯Cl	10.6
C⋯H/H⋯C	9.0
C⋯C	9.9
Se⋯H/H⋯Se	4.2
Se⋯C/C⋯Se	3.0
C⋯Cl/Cl⋯C	3.0
C⋯O/O⋯C	2.6
Cl⋯O/O⋯Cl	2.5
Se⋯Cl/Cl⋯Se	0.6
Se⋯O/O⋯Se	0.5

**Table 4:** Summary of Se⋯O distances (Å) and C—Se—C bond angles (°) in (I)–(IV)

Compound	Se⋯O	C—Se—C	Ref.
(I)	2.8122 (13)	95.62 (8)	this work
(II)	2.7429 (18)	98.43 (12)	Traar *et al.* (2004 ▸)
(III)	2.8760 (12)	98.16 (8)	Woodward *et al.* (2010 ▸)
(IV)	2.8606 (19)	97.41 (12)	McDonagh *et al.* (2016 ▸)

**Table 5:** Experimental details

Crystal data	
Chemical formula	C_19_H_13_ClO_3_Se
*M*_r_	403.70
Crystal system, space group	Monoclinic, *P*2_1_/*c*
Temperature (K)	293
*a*, *b*, *c* (Å)	7.3757 (3), 13.7306 (5), 16.4473 (6)
β (°)	100.002 (1)
*V* (Å^3^)	1640.35 (11)
*Z*	4
Radiation type	Mo *K*α
μ (mm^−1^)	2.47
Crystal size (mm)	0.40 × 0.33 × 0.27

Data collection	
Diffractometer	Bruker APEXII CCD
Absorption correction	Multi-scan (*SADABS*; Sheldrick, 1996 ▸)
*T*_min_, *T*_max_	0.484, 0.745
No. of measured, independent and observed [*I* > 2σ(*I*)] reflections	38518, 3367, 2984
*R*_int_	0.031
(sin θ/λ)_max_ (Å^−1^)	0.626

Refinement	
*R*[*F* ^2^ > 2σ(*F* ^2^)], *wR*(*F* ^2^), *S*	0.026, 0.068, 1.03
No. of reflections	3367
No. of parameters	217
H-atom treatment	H-atom parameters constrained
Δρ_max_, Δρ_min_ (e Å^−3^)	0.36, −0.39

Computer programs: *APEX2* and *SAINT* (Bruker, 2009 ▸), *SIR2014* (Burla *et al.*, 2015 ▸), *SHELXL2014* (Sheldrick, 2015 ▸), *ORTEP-3 for Windows* (Farrugia, 2012 ▸), *DIAMOND* (Brandenburg, 2006 ▸) and *publCIF* (Westrip, 2010 ▸).

## supplementary crystallographic information

### Crystal data

**Table:**

C_19_H_13_ClO_3_Se	*F*(000) = 808
*M**_r_* = 403.70	*D*_x_ = 1.635 Mg m^−^^3^
Monoclinic, *P*2_1_/*c*	Mo *K*α radiation, λ = 0.71073 Å
*a* = 7.3757 (3) Å	Cell parameters from 9049 reflections
*b* = 13.7306 (5) Å	θ = 2.5–26.3°
*c* = 16.4473 (6) Å	µ = 2.47 mm^−^^1^
β = 100.002 (1)°	*T* = 293 K
*V* = 1640.35 (11) Å^3^	Irregular, colourless
*Z* = 4	0.40 × 0.33 × 0.27 mm

### Data collection

**Table:**

Bruker APEXII CCD diffractometer	2984 reflections with *I* > 2σ(*I*)
φ and ω scans	*R*_int_ = 0.031
Absorption correction: multi-scan (SADABS; Sheldrick, 1996)	θ_max_ = 26.4°, θ_min_ = 1.9°
*T*_min_ = 0.484, *T*_max_ = 0.745	*h* = −9→9
38518 measured reflections	*k* = −17→17
3367 independent reflections	*l* = −20→20

### Refinement

**Table:**

Refinement on *F*^2^	0 restraints
Least-squares matrix: full	Hydrogen site location: inferred from neighbouring sites
*R*[*F*^2^ > 2σ(*F*^2^)] = 0.026	H-atom parameters constrained
*wR*(*F*^2^) = 0.068	*w* = 1/[σ^2^(*F*_o_^2^) + (0.0329*P*)^2^ + 0.739*P*] where *P* = (*F*_o_^2^ + 2*F*_c_^2^)/3
*S* = 1.03	(Δ/σ)_max_ = 0.001
3367 reflections	Δρ_max_ = 0.36 e Å^−^^3^
217 parameters	Δρ_min_ = −0.39 e Å^−^^3^

### Special details

**Table:**

Geometry. All esds (except the esd in the dihedral angle between two l.s. planes) are estimated using the full covariance matrix. The cell esds are taken into account individually in the estimation of esds in distances, angles and torsion angles; correlations between esds in cell parameters are only used when they are defined by crystal symmetry. An approximate (isotropic) treatment of cell esds is used for estimating esds involving l.s. planes.

### Fractional atomic coordinates and isotropic or equivalent isotropic displacement parameters (Å^2^)

**Table:**

	*x*	*y*	*z*	*U*_iso_*/*U*_eq_	
Se1	0.50950 (3)	0.20340 (2)	0.19075 (2)	0.05076 (8)	
Cl1	0.74491 (12)	0.48216 (7)	−0.08524 (6)	0.0988 (3)	
O1	0.14227 (17)	0.24860 (9)	0.12331 (8)	0.0427 (3)	
O2	−0.2776 (2)	−0.07713 (12)	0.09235 (12)	0.0741 (5)	
O3	−0.0776 (2)	−0.03054 (10)	0.24246 (10)	0.0628 (4)	
C1	0.2739 (3)	0.26958 (13)	0.19618 (12)	0.0429 (4)	
H9	0.2967	0.3399	0.1975	0.051*	
C2	0.0406 (2)	0.16654 (12)	0.12272 (11)	0.0362 (4)	
C3	−0.0687 (2)	0.14639 (13)	0.04065 (11)	0.0389 (4)	
C4	−0.1805 (2)	0.06325 (14)	0.02911 (12)	0.0432 (4)	
C5	−0.2841 (3)	0.04307 (17)	−0.04809 (13)	0.0563 (5)	
H4	−0.3567	−0.0127	−0.0558	0.068*	
C6	−0.2792 (3)	0.1060 (2)	−0.11328 (13)	0.0644 (6)	
H3	−0.3499	0.0930	−0.1647	0.077*	
C7	−0.1698 (3)	0.18762 (19)	−0.10224 (13)	0.0601 (6)	
H2	−0.1675	0.2297	−0.1464	0.072*	
C8	−0.0625 (3)	0.20824 (16)	−0.02611 (12)	0.0491 (5)	
H1	0.0129	0.2630	−0.0197	0.059*	
C9	−0.1868 (2)	−0.00345 (14)	0.09908 (13)	0.0468 (4)	
C10	−0.0715 (3)	0.02303 (13)	0.18356 (12)	0.0431 (4)	
C11	0.0400 (2)	0.10986 (13)	0.19024 (11)	0.0387 (4)	
C12	0.1505 (3)	0.13595 (14)	0.27303 (11)	0.0479 (4)	
H7	0.2611	0.0965	0.2838	0.057*	
H8	0.0787	0.1232	0.3160	0.057*	
C13	0.2018 (3)	0.24304 (14)	0.27349 (12)	0.0502 (5)	
H6	0.0945	0.2824	0.2774	0.060*	
H5	0.2952	0.2568	0.3214	0.060*	
C14	0.5784 (2)	0.28761 (13)	0.10819 (12)	0.0433 (4)	
C15	0.7139 (3)	0.35717 (15)	0.13040 (13)	0.0508 (5)	
H13	0.7709	0.3629	0.1853	0.061*	
C16	0.7646 (3)	0.41802 (17)	0.07156 (16)	0.0599 (6)	
H12	0.8567	0.4642	0.0862	0.072*	
C17	0.6774 (3)	0.40953 (17)	−0.00896 (15)	0.0587 (5)	
C18	0.5413 (3)	0.3415 (2)	−0.03203 (14)	0.0617 (6)	
H11	0.4824	0.3374	−0.0867	0.074*	
C19	0.4930 (3)	0.27935 (17)	0.02638 (13)	0.0527 (5)	
H10	0.4034	0.2320	0.0110	0.063*	

### Atomic displacement parameters (Å^2^)

**Table:**

	*U*^11^	*U*^22^	*U*^33^	*U*^12^	*U*^13^	*U*^23^
Se1	0.04595 (13)	0.04744 (13)	0.05540 (14)	0.00547 (8)	−0.00095 (9)	0.00375 (9)
Cl1	0.0924 (5)	0.1005 (6)	0.1134 (6)	0.0119 (4)	0.0452 (5)	0.0443 (5)
O1	0.0412 (6)	0.0388 (7)	0.0471 (7)	−0.0011 (5)	0.0046 (5)	0.0103 (6)
O2	0.0652 (10)	0.0486 (9)	0.0975 (13)	−0.0140 (8)	−0.0167 (9)	0.0100 (9)
O3	0.0868 (11)	0.0421 (8)	0.0603 (9)	−0.0118 (7)	0.0148 (8)	0.0101 (7)
C1	0.0477 (10)	0.0305 (8)	0.0495 (10)	0.0001 (7)	0.0056 (8)	−0.0003 (7)
C2	0.0341 (8)	0.0329 (8)	0.0423 (9)	0.0067 (7)	0.0089 (7)	0.0021 (7)
C3	0.0326 (8)	0.0434 (10)	0.0416 (9)	0.0118 (7)	0.0088 (7)	0.0006 (7)
C4	0.0345 (9)	0.0446 (10)	0.0495 (10)	0.0117 (7)	0.0048 (7)	−0.0050 (8)
C5	0.0427 (10)	0.0635 (13)	0.0594 (13)	0.0102 (9)	0.0000 (9)	−0.0145 (11)
C6	0.0501 (12)	0.0963 (19)	0.0437 (11)	0.0186 (13)	−0.0001 (9)	−0.0120 (12)
C7	0.0515 (12)	0.0891 (17)	0.0410 (11)	0.0183 (12)	0.0114 (9)	0.0091 (11)
C8	0.0411 (10)	0.0638 (13)	0.0441 (10)	0.0113 (9)	0.0120 (8)	0.0081 (9)
C9	0.0374 (9)	0.0356 (9)	0.0653 (12)	0.0056 (8)	0.0027 (8)	0.0003 (9)
C10	0.0470 (10)	0.0315 (9)	0.0522 (11)	0.0048 (7)	0.0124 (8)	0.0033 (8)
C11	0.0433 (9)	0.0320 (9)	0.0412 (9)	0.0048 (7)	0.0085 (7)	0.0017 (7)
C12	0.0645 (12)	0.0391 (10)	0.0395 (10)	−0.0009 (9)	0.0076 (9)	0.0026 (8)
C13	0.0664 (13)	0.0386 (10)	0.0458 (10)	−0.0005 (9)	0.0105 (9)	−0.0047 (8)
C14	0.0349 (9)	0.0438 (10)	0.0498 (10)	0.0029 (7)	0.0030 (8)	−0.0071 (8)
C15	0.0432 (10)	0.0541 (12)	0.0536 (11)	−0.0039 (9)	0.0042 (9)	−0.0153 (9)
C16	0.0515 (12)	0.0505 (12)	0.0804 (16)	−0.0078 (10)	0.0189 (11)	−0.0138 (11)
C17	0.0529 (12)	0.0560 (13)	0.0719 (14)	0.0120 (10)	0.0240 (11)	0.0099 (11)
C18	0.0489 (12)	0.0858 (17)	0.0489 (12)	0.0057 (11)	0.0041 (9)	0.0033 (11)
C19	0.0401 (10)	0.0636 (13)	0.0514 (11)	−0.0060 (9)	−0.0006 (9)	−0.0093 (10)

### Geometric parameters (Å, º)

**Table:**

Se1—C14	1.918 (2)	C7—H2	0.9300
Se1—C1	1.9769 (19)	C8—H1	0.9300
Cl1—C17	1.742 (2)	C9—C10	1.541 (3)
O1—C2	1.353 (2)	C10—C11	1.442 (3)
O1—C1	1.434 (2)	C11—C12	1.504 (3)
O2—C9	1.208 (2)	C12—C13	1.518 (3)
O3—C10	1.223 (2)	C12—H7	0.9700
C1—C13	1.505 (3)	C12—H8	0.9700
C1—H9	0.9800	C13—H6	0.9700
C2—C11	1.357 (2)	C13—H5	0.9700
C2—C3	1.473 (2)	C14—C15	1.385 (3)
C3—C8	1.395 (3)	C14—C19	1.388 (3)
C3—C4	1.401 (3)	C15—C16	1.379 (3)
C4—C5	1.392 (3)	C15—H13	0.9300
C4—C9	1.478 (3)	C16—C17	1.373 (3)
C5—C6	1.382 (3)	C16—H12	0.9300
C5—H4	0.9300	C17—C18	1.376 (3)
C6—C7	1.375 (4)	C18—C19	1.377 (3)
C6—H3	0.9300	C18—H11	0.9300
C7—C8	1.389 (3)	C19—H10	0.9300
			
C14—Se1—C1	95.62 (8)	C11—C10—C9	118.81 (16)
C2—O1—C1	117.85 (13)	C2—C11—C10	119.65 (17)
O1—C1—C13	111.73 (16)	C2—C11—C12	121.77 (17)
O1—C1—Se1	110.03 (12)	C10—C11—C12	118.57 (16)
C13—C1—Se1	111.65 (13)	C11—C12—C13	109.32 (15)
O1—C1—H9	107.7	C11—C12—H7	109.8
C13—C1—H9	107.7	C13—C12—H7	109.8
Se1—C1—H9	107.7	C11—C12—H8	109.8
O1—C2—C11	123.53 (16)	C13—C12—H8	109.8
O1—C2—C3	112.17 (14)	H7—C12—H8	108.3
C11—C2—C3	124.29 (16)	C1—C13—C12	110.77 (16)
C8—C3—C4	119.21 (18)	C1—C13—H6	109.5
C8—C3—C2	121.29 (17)	C12—C13—H6	109.5
C4—C3—C2	119.49 (16)	C1—C13—H5	109.5
C5—C4—C3	120.19 (19)	C12—C13—H5	109.5
C5—C4—C9	120.03 (19)	H6—C13—H5	108.1
C3—C4—C9	119.78 (17)	C15—C14—C19	119.8 (2)
C6—C5—C4	119.9 (2)	C15—C14—Se1	119.79 (15)
C6—C5—H4	120.0	C19—C14—Se1	120.39 (15)
C4—C5—H4	120.0	C16—C15—C14	120.3 (2)
C7—C6—C5	120.1 (2)	C16—C15—H13	119.9
C7—C6—H3	120.0	C14—C15—H13	119.9
C5—C6—H3	120.0	C17—C16—C15	119.1 (2)
C6—C7—C8	121.0 (2)	C17—C16—H12	120.4
C6—C7—H2	119.5	C15—C16—H12	120.4
C8—C7—H2	119.5	C16—C17—C18	121.4 (2)
C7—C8—C3	119.6 (2)	C16—C17—Cl1	120.10 (19)
C7—C8—H1	120.2	C18—C17—Cl1	118.46 (19)
C3—C8—H1	120.2	C17—C18—C19	119.6 (2)
O2—C9—C4	122.67 (19)	C17—C18—H11	120.2
O2—C9—C10	119.38 (19)	C19—C18—H11	120.2
C4—C9—C10	117.95 (16)	C18—C19—C14	119.8 (2)
O3—C10—C11	122.40 (18)	C18—C19—H10	120.1
O3—C10—C9	118.79 (17)	C14—C19—H10	120.1
			
C2—O1—C1—C13	−38.2 (2)	O2—C9—C10—C11	178.44 (18)
C2—O1—C1—Se1	86.38 (16)	C4—C9—C10—C11	−1.4 (2)
C1—O1—C2—C11	8.4 (2)	O1—C2—C11—C10	−179.37 (15)
C1—O1—C2—C3	−171.67 (14)	C3—C2—C11—C10	0.7 (3)
O1—C2—C3—C8	−0.4 (2)	O1—C2—C11—C12	1.8 (3)
C11—C2—C3—C8	179.55 (17)	C3—C2—C11—C12	−178.14 (16)
O1—C2—C3—C4	179.30 (14)	O3—C10—C11—C2	−179.78 (18)
C11—C2—C3—C4	−0.8 (2)	C9—C10—C11—C2	0.4 (3)
C8—C3—C4—C5	−0.1 (3)	O3—C10—C11—C12	−0.9 (3)
C2—C3—C4—C5	−179.83 (16)	C9—C10—C11—C12	179.28 (16)
C8—C3—C4—C9	179.35 (16)	C2—C11—C12—C13	18.3 (3)
C2—C3—C4—C9	−0.3 (2)	C10—C11—C12—C13	−160.59 (17)
C3—C4—C5—C6	−1.0 (3)	O1—C1—C13—C12	57.9 (2)
C9—C4—C5—C6	179.49 (18)	Se1—C1—C13—C12	−65.84 (19)
C4—C5—C6—C7	1.0 (3)	C11—C12—C13—C1	−46.3 (2)
C5—C6—C7—C8	0.2 (3)	C19—C14—C15—C16	−0.2 (3)
C6—C7—C8—C3	−1.4 (3)	Se1—C14—C15—C16	179.92 (16)
C4—C3—C8—C7	1.3 (3)	C14—C15—C16—C17	0.9 (3)
C2—C3—C8—C7	−178.99 (17)	C15—C16—C17—C18	−0.3 (3)
C5—C4—C9—O2	1.0 (3)	C15—C16—C17—Cl1	−177.47 (16)
C3—C4—C9—O2	−178.48 (19)	C16—C17—C18—C19	−1.0 (3)
C5—C4—C9—C10	−179.15 (16)	Cl1—C17—C18—C19	176.26 (17)
C3—C4—C9—C10	1.4 (2)	C17—C18—C19—C14	1.6 (3)
O2—C9—C10—O3	−1.4 (3)	C15—C14—C19—C18	−1.1 (3)
C4—C9—C10—O3	178.77 (17)	Se1—C14—C19—C18	178.81 (16)

### Hydrogen-bond geometry (Å, º)

Cg1 is the centroid of the C14–C19 ring.

**Table:**

*D*—H···*A*	*D*—H	H···*A*	*D*···*A*	*D*—H···*A*
C13—H6···O3^i^	0.97	2.59	3.239 (2)	125
C17—Cl1···*Cg*1^ii^	1.74 (1)	3.72 (1)	4.000 (2)	86 (1)

Symmetry codes: (i) −*x*, *y*+1/2, −*z*+1/2; (ii) −*x*+1, −*y*+1, −*z*.
